# The application of extracellular vesicles in colorectal cancer metastasis and drug resistance: recent advances and trends

**DOI:** 10.1186/s12951-023-01888-1

**Published:** 2023-04-29

**Authors:** Linjin Xiong, Yumeng Wei, Qiang Jia, Jinglin Chen, Tao Chen, Jiyuan Yuan, Chao Pi, Huiyang Liu, Jia Tang, Suyu Yin, Ying Zuo, Xiaomei Zhang, Furong Liu, Hongru Yang, Ling Zhao

**Affiliations:** 1 Key Laboratory of Medical Electrophysiology, Ministry of Education, School of Pharmacy of Southwest, Medical University, Luzhou, 646000 People’s Republic of China; 2 Key Laboratory of Medical Electrophysiology, Ministry of Education, The Affiliated Traditional Chinese Medicine Hospital of Southwest Medical University, No.182, Chunhui Road, Longmatan District, Luzhou, 646000 Sichuan People’s Republic of China; 3 Central Nervous System Drug Key Laboratory of Sichuan Province, School of Pharmacy of Southwest, Medical University, Luzhou, 646000 Sichuan People’s Republic of China; 4 Luzhou Key Laboratory of Traditional Chinese Medicine for Chronic Diseases Jointly Built by Sichuan and Chongqing, The Affiliated Traditional Chinese Medicine Hospital of Southwest Medical University, Luzhou, 646000 Sichuan People’s Republic of China; 5 Ethics Committee Office, The Affiliated Traditional Chinese Medicine Hospital of Southwest Medical University, Luzhou, 646000 Sichuan China; 6 Clinical Trial Center, The Affiliated Traditional Chinese Medicine Hospital of Southwest Medical University, Luzhou, 646000 Sichuan People’s Republic of China; 7 Department of Comprehensive Medicine, The Affiliated Traditional Chinese Medicine Hospital of Southwest Medical University, Luzhou, 646000 Sichuan China; 8 Luzhou Key Laboratory of Traditional Chinese Medicine for Chronic Diseases Jointly Built by Sichuan and Chongqing, Institute of Medicinal Chemistry of Chinese Medicine, Chongqing Academy of Chinese Materia Medica, Chongqing, 400065 People’s Republic of China; 9 Department of Oncology, The Affiliated Traditional Chinese Medicine Hospital of Southwest Medical University, No.182, Chunhui Road, Longmatan District, Luzhou, 646000 Sichuan China; 10 Department of Oncology, The Affiliated Hospital of Southwest Medical University, Luzhou, 646000 Sichuan China

**Keywords:** Extracellular vesicles, Colorectal cancer, Metastasis, Drug resistance, Drug delivery system, Biomarkers

## Abstract

Colorectal cancer (CRC) has high incidence and mortality rates and is one of the most common cancers of the digestive tract worldwide. Metastasis and drug resistance are the main causes of cancer treatment failure. Studies have recently suggested extracellular vesicles (EVs) as a novel mechanism for intercellular communication. They are vesicular particles, which are secreted and released into biological fluids, such as blood, urine, milk, etc., by a variety of cells and carry numerous biologically active molecules, including proteins, nucleic acids, lipids, metabolites, etc. EVs play a crucial part in the metastasis and drug resistance of CRC by delivering cargo to recipient cells and modulating their behavior. An in-depth exploration of EVs might facilitate a comprehensive understanding of the biological behavior of CRC metastasis and drug resistance, which might provide a basis for developing therapeutic strategies. Therefore, considering the specific biological properties of EVs, researchers have attempted to explore their potential as next-generation delivery systems. On the other hand, EVs have also been demonstrated as biomarkers for the prediction, diagnosis, and presumed prognosis of CRC. This review focuses on the role of EVs in regulating the metastasis and chemoresistance of CRC. Moreover, the clinical applications of EVs are also discussed.

**Graphical Abstract:**

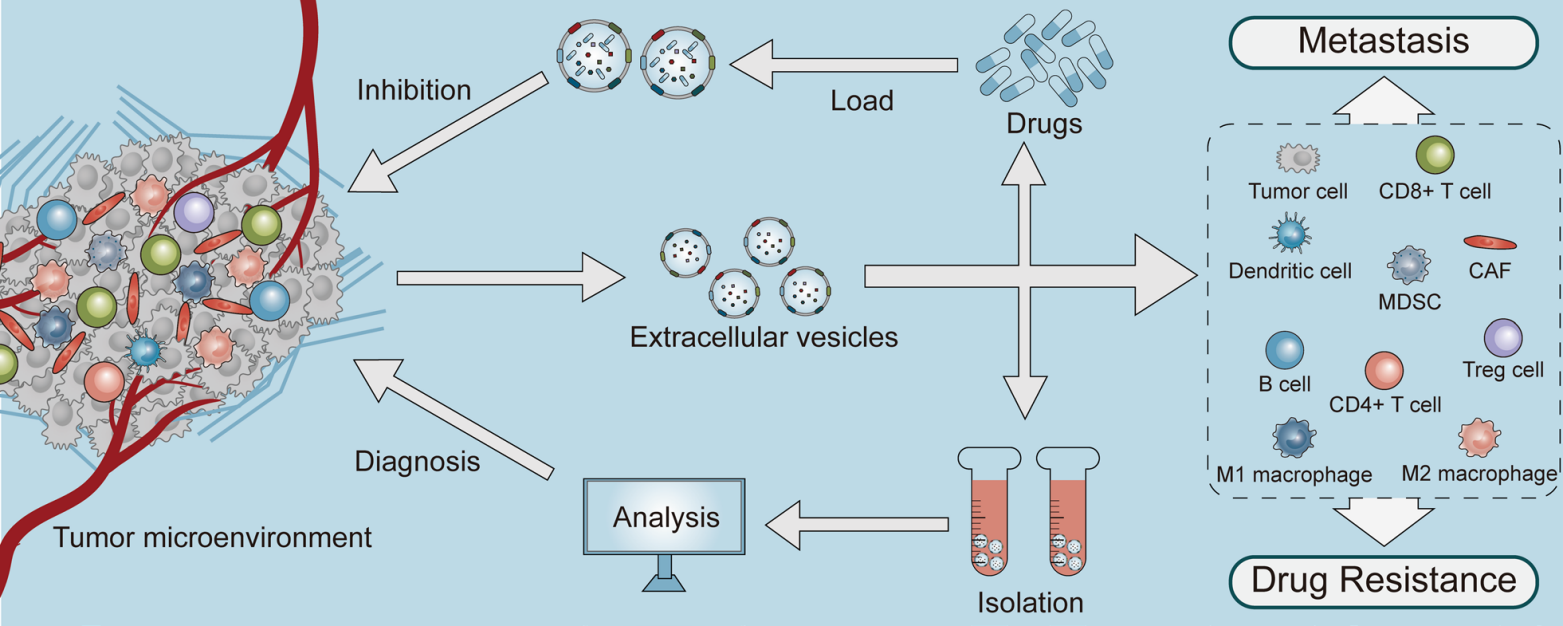

## Introduction

Colorectal cancer (CRC) is the third most deadly cancer worldwide, with high incidence and mortality [1]. Globally, approximately 10.0% of cancer incidence and 9.4% of cancer-related mortality are caused by CRC, and nearly 900,000 people die from CRC each year [1, 2]. Smoking, alcohol intake, low intake of vegetables and fruits, and excessive obesity are high-risk factors for the development of CRC [2]. Metastasis and drug resistance are major challenges for CRC treatment.

**Fig. 1:**
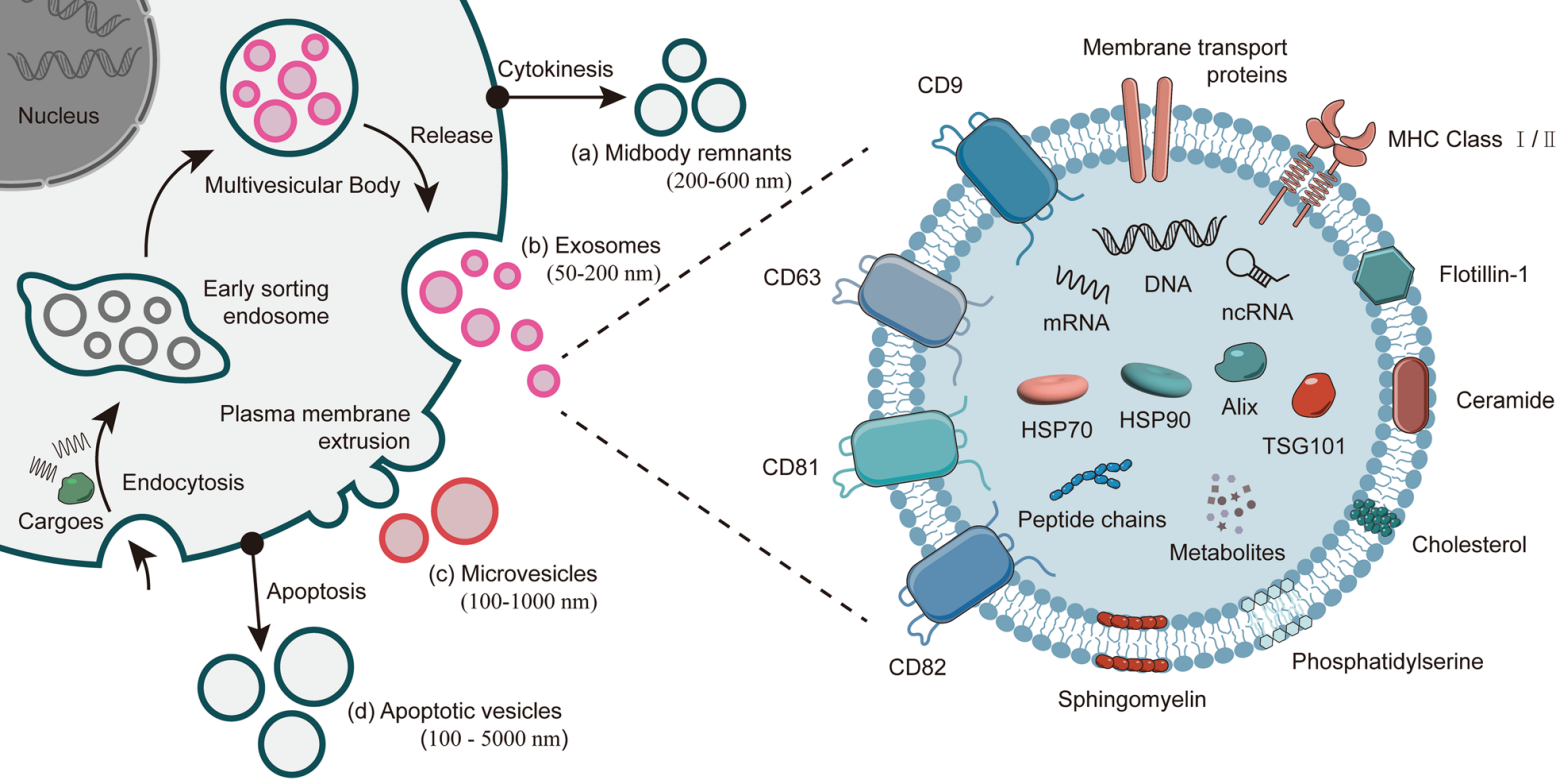
EVs biogenesis and structure. **a** the shed midbody remnants are released during cytoplasmic division and have a diameter, ranging from 200 to 600 nm; **b** exosomes are the smallest EV subpopulation, produced by the fusion of MVBs with the cell surface and the release of intraluminal vesicles (ILVs), with a size of 50–200 nm; **c** microvesicles are larger than exosomes, formed by the direct outward budding of the plasma membrane, with a diameter range of 100–1000 nm. **d** apoptotic vesicles are found during apoptosis with sizes, ranging from 100 to 5000 nm [4, 5]

Recent studies have explored the regulatory pathways associated with extracellular vesicles (EVs) in CRC metastasis and drug resistance. EVs are a collective term, covering a variety of cell-released subtypes of membrane structures [3]. Since their contents, membrane composition, and size depend on their cell of origin and mode of biogenesis, EVs are a highly heterogeneous assemblage of subpopulations [3]. Despite their high heterogeneity, EVs can be classified based on the biogenesis patterns and characteristics into four subtypes, including exosomes, microvesicles, midbody remnants, and apoptotic vesicles (Fig. 1). For a clearer discussion, this review has focused on the EVs subtypes formed by interaction with the plasma membrane: exosomes, and microvesicles.

**Fig. 2:**
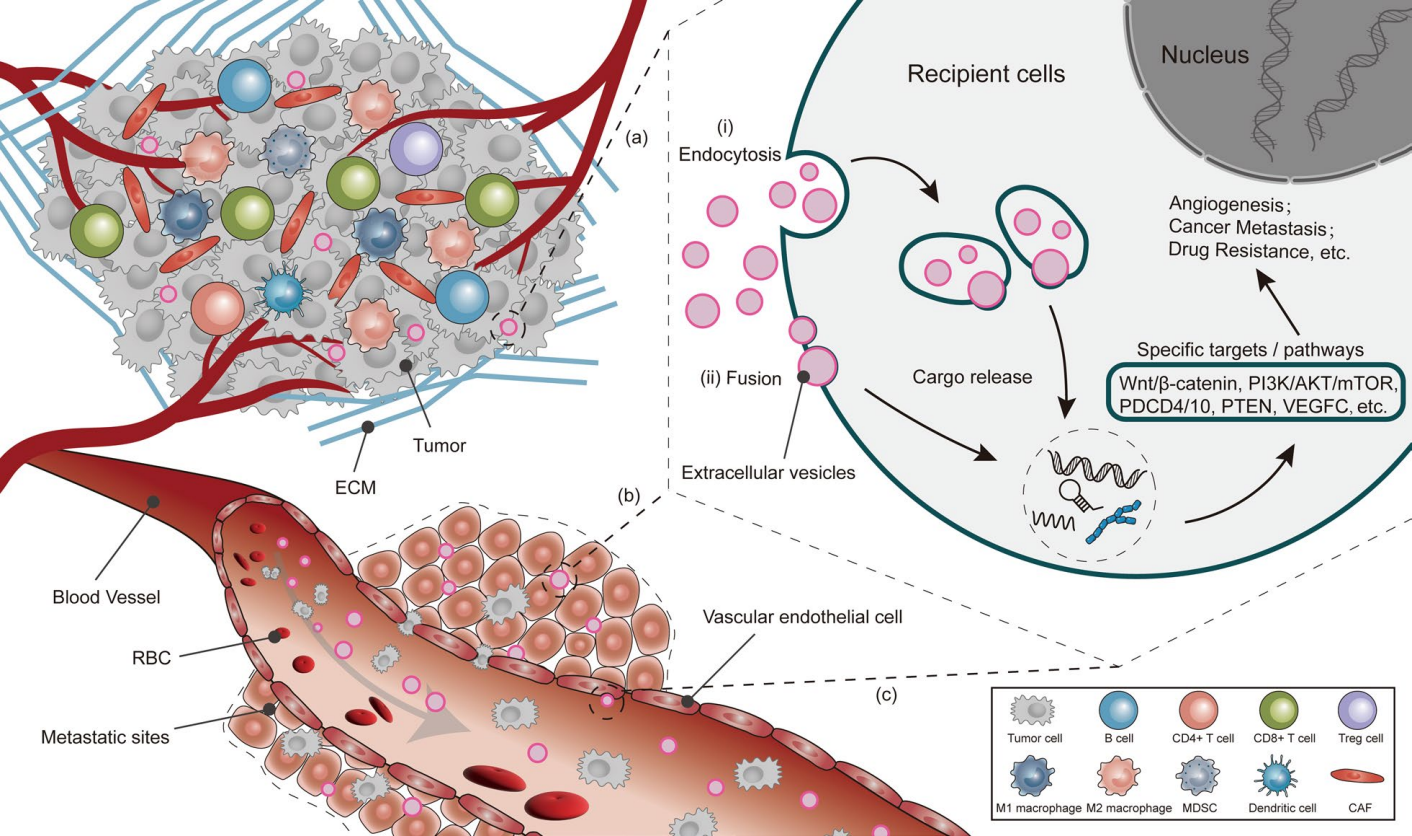
Diagram of EVs transport. EVs secreted by tumor and stromal cells: **a** captured in CRC tissues; **b** metastasized to metastatic sites via the blood circulation; **c** captured by vascular endothelial cells. EVs undergo (i) endocytosis or (ii) fusion with the recipient cells and release bioactive substances to activate signaling, ultimately leading to CRC metastasis and drug resistance

EVs are phospholipid bilayer-based substances, which are found in a variety of bodily fluids, including blood, urine, and milk. They contain a large number of proteins, nucleic acids, lipids, and metabolites (Fig. 1) [6, 7]. And they can be obtained by differential centrifugation, density-gradient centrifugation, sucrose buffered centrifugation, affinity capture, gel-permeation chromatography, precipitation, microfluidic devices, and membrane filtration separations [8]. In terms of structure and composition (Fig. 1), the protein components in EVs include (a) membrane transport and fusion-related proteins, such as annexin, Rab-GTPase, and heat shock proteins (HSP); (b) tetraspanins, such as CD9, CD63, CD81, CD82, and intercellular cell adhesion molecule (ICAM) -1; (c) multivesicular bodies (MVBs) -related proteins, including ALIX and TSG101; and (d) other proteins, such as integrins [9]. All these proteins are essential for the EVs function. The lipid components in EVs include ceramide, cholesterol, phosphatidylserine, and sphingomyelin [10]. Notably, the CD9, CD63, CD81, CD82, endosomal sorting complex required for transport (ESCRT) proteins (TSG101 and ALIX), flotillin, ceramide, major histocompatibility complex (MHC), and HSP70/90 have been identified as potential markers for the subgroups of EVs [11]. Functionally, EVs are involved in many physiological and pathological processes. Their biological functions mainly depend on the transfer of active substances, such as non-coding RNAs, proteins, and nucleic acids, to nearby or distant receptor cells, thereby altering the behavior of receptor cells. In CRC, EVs can regulate the function and phenotype of recipient cells by transferring the cancer-associated cargoes, thereby contributing to the alteration of the microenvironment surrounding the CRC cells and enhancing their viability (Fig. 2) [12]. In addition, due to their specific biological properties, EVs are considered a biomarker for the prediction, diagnosis, and presumed prognosis of CRC. Furthermore, researchers have invested great efforts to explore their potential as a next-generation delivery system. This review focuses on the role of EVs in regulating the metastasis and chemoresistance of CRC and discusses the strategies for the clinical applications of EVs.

## Extracellular vesicles in colorectal cancer

**Fig. 3:**
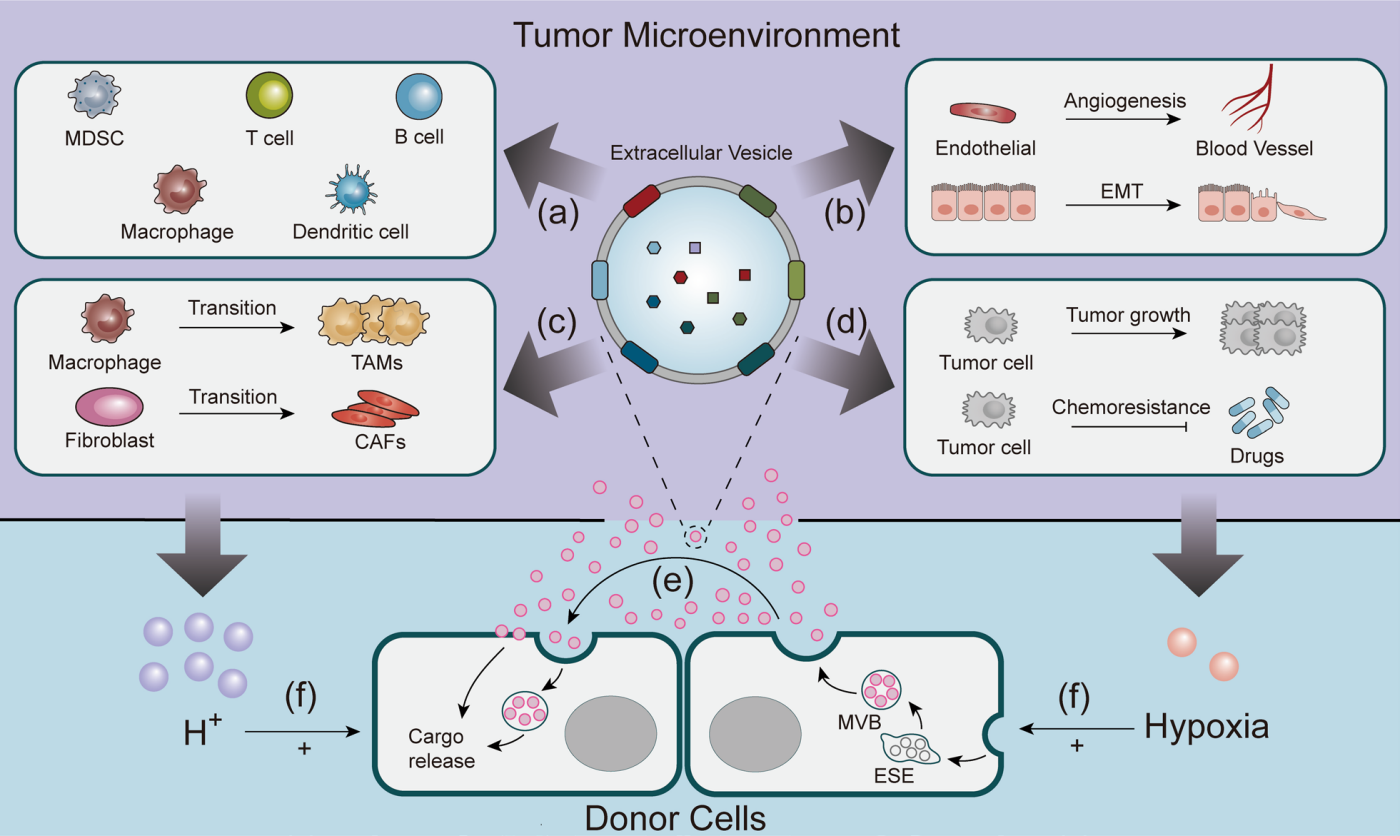
The role of EVs in CRC. Donor cells secrete EVs into TME: **a** EVs act on immune cells to regulate immunosuppression; **b** EVs are taken up by recipient cells to promote angiogenesis and EMT; **c** EVs induce macrophages and fibroblasts transformation to favor CRC growth; **d** EVs are taken up by CRC cells to promote proliferation and chemoresistance; **e** EVs are also taken up by donor cells to exert some effect; **f** the microacidic and hypoxic environment in TME could accelerate the release of EVs from donor cells

EVs are a significant signaling tool in the tumor microenvironment (TME). They induce the TME around CRC cells to be reprogrammed, thereby conferring immunity to CRC cells against eradication and promoting CRC progression (Fig. 3). Fibroblasts can take up the CRC cell-derived exosomes (CEXs) and transform into cancer-associated fibroblasts (CAFs). The activated CAFs can still take up the EVs from CRC cells and undergo lipid metabolic reprogramming, which promotes the secretion of EVs through the CXCL5-CXCR2 axis and forms a positive-feedback loop, thereby aiding in the CRC growth and metastasis [13].

The CRC cells are often in a hypoxic and slightly acidic microenvironment, which might be due to increased oxygen consumption, reduced oxygen entry, and difficulty in eliminating metabolic wastes from cancer cells [14]. This host microenvironment helps CRC cells in evading immune surveillance and resisting the cytotoxicity of chemotherapeutic agents. Under these hypoxic and slightly acidic microenvironments, the CRC cells accelerate the release of EVs [15]. Moreover, in order to take nutrients from the host, the CRC tissues form more ducts to transport these “spoils”. Consequently, these abundantly secreted EVs are taken up by endothelial cells, promoting vascular sprouting to meet the growing needs of the CRC tissues. Studies showed that the CRC cell-derived exosomal miR-1229 could promote angiogenesis by targeting *HIPK2* and inhibiting its protein expression, thereby activating the VEGF signaling pathway [16]. In addition, a study demonstrated that the horizontal transfer of B cell-derived CD19^+^ EVs to the chemotherapy-treated cancer cells, followed by ATP hydrolysis to adenosine via the CD39 and CD73 vesicle-binding proteins could inhibit the CD8^+^ T cell responses and promote immunosuppression [17].

These findings provided a novel insight that the EVs in the TME could carry active molecules, which directly regulated receptor cells; moreover, this effect occurred without direct contact between cells. This might greatly improve the efficiency of communication because a large number of EVs derived from a single cell might be taken up by multiple cells, leading to the activation of multiple pathways and alteration of regulated receptor cells; moreover, this also illustrated the important regulatory role of EVs in the CRC microenvironment. The role of EVs in CRC metastasis and drug resistance has been discussed in detail in the following sections.

## Extracellular vesicles regulate metastasis in colorectal cancer

**Fig. 4:**
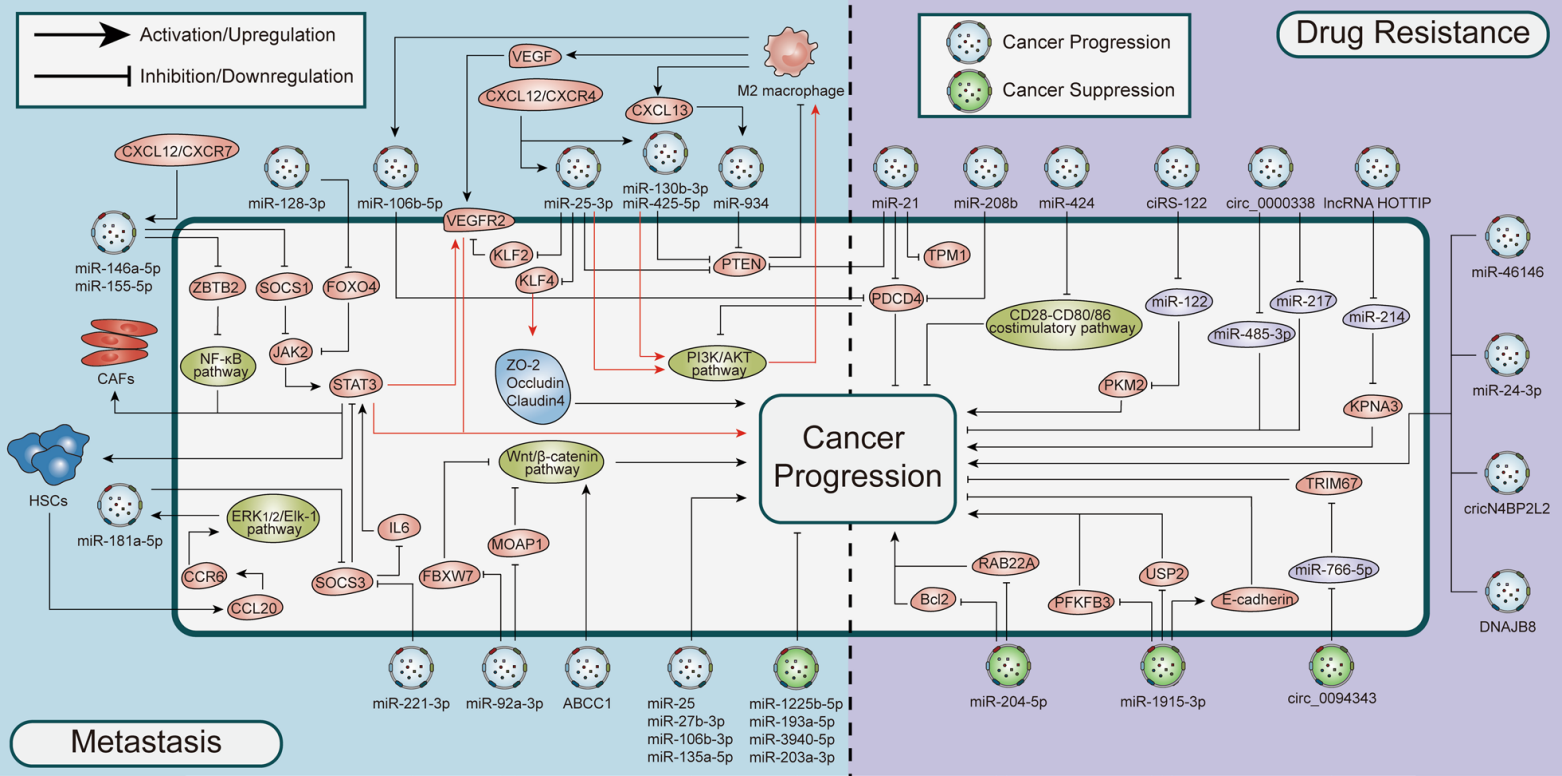
EVs regulate metastasis and drug resistance in CRC. Molecular signaling pathways of metastasis and drug resistance, including PI3K, Akt, phosphatase and tensin homolog (PTEN), STAT3, programmed cell death 4 (PDCD4), Wnt, β-catenin, and suppressor of cytokine signaling 3 (SOCS3), are regulated by EVs. Induction of metastasis and drug resistance promotes CRC progression, and targeting EVs may impair CRC growth

Distant metastasis is a major cause of cancer-related deaths in CRC patients. Approximately, 15–25% of CRC patients have liver metastases at the time of initial diagnosis [18]. This section summarizes the role of EVs in the invasion and metastasis of CRC. Table 1 summarizes some EV cargos related to CRC metastasis. The molecular mechanisms by which different EVs regulate CRC metastasis are shown in Fig. 4.

**Table 1:** Role of cargoes in EVs in regulating metastasis in CRC

non-coding RNA/protein/gene	Source	Expression pattern	Action target/pathway	Outcome	References
a. Promote cancer progression					
miR-27b-3p	Tumor	Upregulated	VE-Cad, p120	Increase vascular permeability; Promote the generation and metastasis of CTCs	[30]
miR-25-3p, miR-130b-3p, miR-425-5p, miR-934	Tumor	Upregulated	KLF2, KLF4, PI3K / Akt, PTEN	Angiogenesis; M2 polarization of macrophages	[29, 96, 97]
miR-146a-5p, miR-155-5p	Tumor	Upregulated	SOCS1, ZBTB2	Promote activation of CAFs; Induction of EMT	[22]
miR-181a-5p	Tumor	Upregulated	SOCS3, IL6/STAT3	Activate HSC; PMN formation	[32]
miRNA-106b-5p, miR-106b-3p	Tumor/serum	Upregulated	PDCD4, DLC-1	M2 polarization of macrophages; Accelerated metastasis; Induction of EMT	[12, 98]
miR-135a-5p, miR-25	Tumor	Upregulated	LATS2 /YAP1 / MMP7, SIRT6	PMN formation; Liver metastases	[18, 99]
miR-221-3p, miR-221/222	Tumor/serum	Upregulated	SOCS3, SPINT1	Angiogenesis; Liver metastases; PMN formation	[28, 100]
miR-128-3p	Tumor	Upregulated	FOXO4, TGF-β / SMAD, JAK / STAT3	Induction of EMT	[20]
miR-17-5p	CAF	Upregulated	RUNX3/ MYC / TGF-β1	Accelerated metastasis	[101]
miR-92a-3p	CAF	Upregulated	Wnt / β-catenin, FBXW7, MOAP1	Inhibit mitochondrial apoptosis to promote cell stemness, EMT, metastasis, and 5-FU / OX resistance	[47]
miR-21-5p, miR-155-5p	TAM	Upregulated	BRG1	Promote CRC cell migration and invasion	[102]
miR-1229	Serum	Upregulated	HIPK2	Angiogenesis; Accelerated metastasis	[16]
circPACRGL	Tumor	Upregulated	miR-142-3p, miR-506-3p	Accelerated metastasis; Promotion of N1 to N2 differentiation of neutrophils	[103]
circLONP2	Tumor	Upregulated	DDX1, DGCR8 / Drosha	Accelerated metastasis	[104]
circ-133	plasma	Upregulated	miR-133a / GEF-H1 / RhoA	Accelerated metastasis	[105]
circ-ABCC1	Tumor	Upregulated	Wnt / β-catenin	Accelerated metastasis	[106]
circEIF3K	CAF	Upregulated	miR-214	Accelerated metastasis	[107]
lncRNA RPPH1, lncRNA HLA-F-AS1	Tumor	Upregulated	TUBB3, miR-375	Induction of EMT; M2 polarization of macrophages; Accelerated metastasis	[108, 109]
lncRNA MALAT1	Tumor	Upregulated	miR-26a/26b, FUT4, PI3K / Akt / mTOR	Accelerated metastasis	[110]
HSPC111	Tumor	Upregulated	ACLY	PMN formation; Liver metastasis	[13]
IRF-2	Tumor	Upregulated	VEGFC	To guide tumor metastasis in SLN	[111]
PrPC	Tumor	Upregulated	-	Angiogenesis; Accelerated metastasis	[112]
Gas6, DPP4	Tumor	Upregulated	Axl, Twist1, Smad pathway	Angiogenesis	[113, 114]
b. Inhibit cancer progression					
miR-1255b-5p	Tumor	Downregulated	hTERT	Inhibit EMT; Inhibit metastasis	[21]
miR-193a-5p	plasma	Downregulated	CUX1, ITSN1	Inhibit metastasis	[88]
miR-3940-5p	MSC	Upregulated	ITGA6	Inhibit EMT; Inhibit metastasis	[115]
miR-203a-3p	Hepatocyte	–	E-cadherin, Src	Inhibit metastasis	[33]
circRHOBTB3	Tumor	Downregulated	ENO1, ENO2	Inhibition of EMT; Tumor suppressor	[116]
ANGPTL1	Tumor	Downregulated	JAK2 / STAT3	Inhibit liver metastasis; Block vascular leakage	[34]

*VE-Cad* vascular endothelial cadherin, *KLF2* Krüppel-like factor 2, *KLF4* Krüppel-like factor 4, *PTEN* Phosphatase and tensin homolog, *SOCS1* suppressor of cytokine signaling 1, *ZBTB2* zinc finger and BTB domain containing 2, *SOCS3* suppressor of cytokine signaling 3, *PDCD4* programmed cell death 4, *DLC-1* deleted in liver cancer-1, *LATS2* large tumor suppressor kinase 2, *YAP1* yes-associated protein 1, *SIRT6* sirtuin 6, *SPINT1* serine protease inhibitor Kunitz type 1, *RUNX3* 3'-untranslated regions (UTRs) of RUNX family transcription factor 3, *DDX1* DEAD-Box 1, *DGCR8* DiGeorge syndrome critical region gene 8, *GEF-H1* Rho guanine nucleotide exchange factor, *TUBB3* β-III tubulin, *FUT4* fucosyltransferase 4, *ACLY* ATP-citrate lyase, *DPP4* dipeptidyl peptidase IV, *PrPC* prion protein, *hTERT* human telomerase reverse transcriptase, *MSC* mesenchymal stem cell, *CUX1* CUT-like homeobox 1, *ITSN1* intersectin 1, *ITGA6* Integrin alpha6, *NFs* normal fibroblasts, *SLN* Sentinel lymph nodes

### Extracellular vesicles in epithelial-mesenchymal transition

Epithelial-to-mesenchymal transition (EMT) is the process, by which, the cancer cells lose their epithelial cell-like characteristics and gain mesenchymal cell-like characteristics. This process is associated with enhanced CRC cell mobility, invasiveness, and resistance to apoptosis [19]. Recently, Bai's team showed that the CRC cell HCT-116-derived CEXs miR-128-3p could promote EMT by downregulating the FOXO4 expression and activating the transforming growth factor-β (TGF-β)/SMAD and JAK/STAT3 signaling pathways [20]. Furthermore, the telomerase contents and stability might affect the process of hypoxia regulation in EMT. A study showed that the decreased secretion of tumor suppressor exosomal miR-1255b-5p in the serum samples obtained from CRC patients could lead to the increased expression of human telomerase reverse transcriptase (hTERT), which might further enhance EMT and telomerase activity by activating the Wnt/β-catenin signaling pathway [21].

CEXs can also induce the activation of non-malignant cells, thereby accelerating the phenotypic changes in CRC cells. For example, CEXs could transfer miR-106b into macrophages and activate PI3Kγ/AKT/mTOR signaling pathway by directly inhibiting the PDCD4 protein, leading to the M2 polarization of macrophages. In turn, the activated M2-type macrophages mediate the EMT and metastasis of CRC cells through a positive feedback approach [12]. Similarly, the CEXs could also transfer miR-146a-5p and miR-155-5p into CAFs by targeting the suppressor of cytokine signaling 1 (SOCS1) and zinc finger and BTB domain-containing 2 (ZBTB2) and activating the JAK2-STAT3/NF-κB signaling pathway, which promoted the activation of CAFs. The activated CAFs showed a significant increase in the expression levels of IL-6, TNF-α, TGF-β, and CXCL12 while promoting EMT in the CRC cells [22].

The E-cadherin knockdown is a critical step in the initiation of EMT. ZEB can inhibit the expression of E-cadherin by binding to its promoter, while Twist can indirectly inhibit E-cadherin activity, ultimately leading to the occurrence of EMT [23]. ZEB1 was identified as a direct target of miR-150-5p. The CRC patients showed a significant decrease in the serum exosomal miR-150-5p levels and upregulation of ZEB1 expression as compared to the control group; this might lead to poor prognosis of CRC patients [24].

### Extracellular vesicles in matrix activation

Multiple cancer signals delivered through EVs might contribute to the formation of an activated stromal and inflammatory microenvironment. The ITGBL (integrin β-like) 1-enriched CEXs can enter the blood circulation and are taken up by resident fibroblasts, inducing the high secretion of pro-inflammatory cytokines, such as IL-6 and IL-8. Further studies revealed that these CEXs could promote the formation of an inflammatory microenvironment by stimulating the TNFAIP3-mediated NF-κB signaling pathway, which ultimately accelerates the metastasis of CRC cells [25]. On the other hand, Hyaluronan and proteoglycan link protein-1 (HAPLN1), an extracellular matrix (ECM) protein, is involved in maintaining a normal colon microenvironment. The CRC patients demonstrated reduced mRNA levels of *HAPLN1*, which could induce the development of CRC [26]. Microfibril-associated glycoprotein (MAGP)-1 is another key protein in the ECM. However, its expression was also found to be down-regulated in CRC patients [27].

### Extracellular vesicles in angiogenesis and vascular permeability

Angiogenesis and altered vascular permeability are the key processes in CRC growth and metastasis, especially in the initial stages of pre-metastatic niches (PMNs) formation. In fact, the serum expression levels of miR-19a-3p, miR-203-3p, miR-221-3p, and let-7f-5p were significantly higher in CRC patients as compared to those in the healthy subjects. Among them, miR-221-3p was transferred by CEXs and regulated the STAT3/VEGFR-2 axis by targeting SOCS3 in endothelial cells to promote the proliferation and migration of endothelial cells and the formation of vascular-like structures [28].

EVs also increase vascular permeability. A study showed that CEXs miR-25-3p could increase angiogenesis and vascular permeability, thereby enhancing the liver and lung metastases of CRC in mice [29]. Specifically, the exosomal miR-25-3p targets KLF2 and KLF4 to regulate the expression levels of endothelial VEGFR2, ZO-1, occludin, and claudin 5, thereby accelerating the CRC progression [29]. Similarly, the EMT-CEXs miR-27b-3p was upregulated in the CRC tissues. Further studies revealed that it could promote the expression of vascular endothelial adhesion protein (VE-Cad) and p120 at the post-transcriptional level by directly binding to the 3'-untranslated region of VE-Cad and p120 to increase the vascular permeability, which accelerated the production and metastasis of circulating CRC cells [30].

As a “double-edged sword”, not all the EVs are involved in the angiogenesis and disruption of vascular permeability. Knocking down the lncRNA-APC1 in exosomes led to the activation of the MAPK pathway in endothelial cells to promote angiogenesis. In contrast, APC inhibited the enrichment of PPARα on the lncRNA-APC1 promoter, leading to the expression of lncRNA-APC1 and inhibition of angiogenesis [31].

### Extracellular vesicles in metastatic niche formation

The resident cells at metastatic sites uptake the EVs released from CRC cells, which results in promoting the formation of PMNs and providing suitable soil for CRC metastasis. Liver is a frequent target for the invasion and metastasis of CRC and might be related to its abundant blood flow and high uptake of EVs in the blood. Zhao and his team found that the miR-181a-5p-enriched CEXs could induce the activation of the hepatic stellate cells (HSCs) by targeting SOCS3 while simultaneously activating the IL6/STAT3 signaling pathway [32]. The activated HSCs secrete the chemokine CCL20, thereby promoting the remodeling of TME and the formation of PMNs [32]. Kupffer cells (KCs) are the main members of the defense system. It was suggested that the KCs could engulf the exosomal miR-135a-5p from the blood into the liver. When these CEXs are trapped, they trigger the formation of PMNs in the liver through the large tumor suppressor kinase 2/yes-associated protein/matrix metalloproteinase (MMP) 7 axis [18]. However, studies have also shown that the EVs might be involved in inhibiting EMT in CRC cells, reprogramming KCs, and reducing the expression of MMP9, thereby preventing vascular leakage and formation of PMNs in the liver [33, 34].

### Extracellular vesicles in immune evasion

The TME of CRC is infiltrated by various innate or adaptive immune cells, including macrophages, neutrophils, dendritic cells (DCs), T lymphocytes, B lymphocytes, and natural killer (NK) cells [14]. In CRC, lymphangiogenesis is regulated by the VEGFC/VEGFR3 signaling pathway. The VEGFR3 signaling pathway in tumor-associated macrophages (TAMs) is activated by the abundant VEGFC in CRC, resulting in the induction of CRC immune escape and acceleration of tumor growth [35]. A study reported that the M2 macrophage-derived exosome miR-155-5p could promote the proliferation and anti-apoptotic activities of SW48 and HT29 cells; it was also confirmed in xenograft tumor models that exosomal miR-155-5p decreased the expression levels of *ZC3H12B* and upregulated IL-6 levels, thereby accelerating the induction of immune escape and CRC occurrence [36]. In addition, the M2 macrophage-derived EVs miR-186-5p promoted CRC growth and motility by targeting and inhibiting *DLC1* expression [37]. Macrophages can take up EVs secreted by CRC cells and undergo M2 polarization and PD-L1 expression, thereby increasing the abundance of PD-L1 CD206 macrophages in the CRC microenvironment and suppressing T cell activity, ultimately leading to immunosuppression [38].

The cancer cells secrete EVs to transfer immunosuppressive molecules, which mediate the metabolic reprogramming of T cells and drive their functional depletion [39]. Meanwhile, EVs could inhibit the clonal expansion capacity of T cells by mediating adenosine production, inhibiting creatine import, and reducing ATP production, which lead to immunosuppression [40, 41]. This might be one of the reasons for the failure of adoptive CAR-T immunotherapy.

Myeloid-derived suppressor cells (MDSCs) are a heterogeneous group of immature myeloid cells with immunosuppressive activities and are induced by a variety of molecules present in the TME, such as VEGF, IFN-γ, IL-1β, IL-4, IL-6, IL-13, and TNF [15]. It was found that the plasma of CRC patients exhibited higher levels of MDSC-derived exosomal S100A9 and enhanced CRC cells stemness and growth [42].

## Extracellular vesicles regulate drug resistance in colorectal cancer

The effective treatment of metastatic cancer often requires the use of drugs. Nevertheless, the development of drug resistance has been a major issue in successful CRC treatment, especially in the late stages of disease progression. This section summarizes the role of EVs in modulating drug resistance in CRC (Fig. 4, Table 2).

**Table 2:** Role of cargoes in EVs in regulating drug resistance in CRC

non-coding RNA/protein/gene	Source	Expression pattern	Action target/pathway	Outcome	References
a. Chemical resistance					
miR-208b	Tumor	Upregulated	PDCD4	OX resistance	[117]
miR-424	Tumor	–	CD28-CD80/86 costimulatory pathway	Immune checkpoint blockade	[44]
miR-46146	Tumor	Upregulated	PDCD10	OX resistance	[118]
miR-21	Tumor	Upregulated	PDCD4, TPM1, PTEN	5-FU resistance	[119]
miR-24-3p	CAF	Upregulated	CDX2	MTX resistance	[56]
ciRS-122	Tumor	Upregulated	miR-122	OX resistance	[120]
circ_0000338	Tumor	Upregulated	miR-217, miR-485-3p	5-FU resistance	[121]
cricN4BP2L2	CAF	Upregulated	EIF4A3	OX resistance	[49]
lncRNA HOTTIP	Tumor	Upregulated	miR-214	Mitomycin resistance	[50]
lncRNA CCAL	CAF	Upregulated	mRNA stabilizing protein HuR	OX resistance	[48]
p-STAT3	Tumor	Upregulated	-	5-FU resistance	[53]
DNAJB8	Tumor	Upregulated	TP53	OX resistance	[51]
b. Chemically sensitive					
miR-1915-3p	FHC	Upregulated	PFKFB3, USP2, E-cadherin	OX sensitive	[75]
miR-204-5p	HEK293T	Upregulated	RAB22A, Bcl2	5-FU sensitive	[122]
circ_0094343	CAF	Downregulated	miR-766-5p	Sensitive to 5-FU, OX, and Dox	[123]

*PDCD4* programmed cell death factor 4, *PDCD10* Programmed cell death 10, *TPM1* Tropomyosin 1, *PTEN* Phosphatase and tensin homolog, *MTX* methotrexate, *CDX2* caudal‐related homeobox 2, *EIF4A3* Eukaryotic initiation factor 4A-III, *5-FU* 5-Fluorouracil, *OX* Oxaliplatin, *PFKFB3* 6-phosphofructo-2-kinase/fructose-2,6-biphosphatase 3, *USP2* ubiquitin carboxyl-terminal hydrolase 2, *Dox* doxorubicin

### Extracellular vesicles in immune resistance

Immunotherapy has become a powerful strategy in cancer treatment. Immune checkpoint inhibitors (ICIs) and adoptive cellular immunotherapy have been clinically used to manipulate the body's immune system and kill cancer cells [43]. However, the robust adaptability of CRC enables them to escape immunotherapy. The analysis of immune profiles and tumor-immune cell interactions in human CRC showed that the miR-424-enriched CEXs were taken up by infiltrating T cells and DCs, leading to the failure of ICI immunotherapy by inhibiting the CD28-CD80/86 co-stimulatory signaling pathway [44]. Ploeg and his team attempted to bypass this EVs-mediated immune resistance to CRC. They developed a novel bispecific antibody CD73xEpCAM, which could bind to the surface marker EpCAM of EVs and inhibit CD73 (GPI-anchored ecto 5'-nucleotidase), thereby effectively enhancing the immune activity [45].

### Extracellular vesicles and signaling pathway activation

The Wnt/β-catenin signaling pathway plays an important role in maintaining intestinal homeostasis. However, sustained activation of the Wnt/β-catenin signaling pathway might lead to the accumulation of β-catenin, thereby promoting CRC [46]. Studies have shown a significant increase in the expression of miR-92a-3p in CRC patients. The internalization of CAF-derived exosomal miR-92a-3p by CRC cells could increase the contents of miR-92a-3p in CRC cells, resulting in the activation of the Wnt/β-catenin signaling pathway. Meanwhile, the miR-92a-3p could also inhibit mitochondrial apoptosis by directly targeting FBXW7 and MOAP1. These processes contribute to enhancing the stemness of CRC cells and promote EMT as well as chemoresistance [47]. In addition, long non-coding RNAs are involved in the development of drug resistance. lncRNA CCAL, acting on the mRNA-stabilizing protein HuR (human antigen R), could increase the mRNA and protein levels of β-catenin, which contributed to promoting oxaliplatin resistance in CRC cells [48].

The PI3K/AKT/mTOR signaling pathway is involved in regulating the proliferation, metabolism, autophagy, and protein and lipid synthesis in the cells. The aberrant activation of the PI3K/AKT/mTOR signaling pathway is frequently observed in cancers, including CRC [46]. Recently, a study reported that the CAF-derived exosomal cricN4BP2L2 promoted the resistance of CRC LoVo cells to oxaliplatin by upregulating EIF4A3, leading to the activation of PI3K/AKT/mTOR signaling pathway, while inhibiting the development of apoptosis [49].

Nowadays, the phenomenon of CRC drug resistance is becoming more prominent. The measures, required to overcome the development of drug resistance, should be explored. Finding molecular targets for CRC therapy based on signaling pathways might be an effective way to discover novel drugs due to their close correlations with CRC function, such as ICIs, which have been used in clinical practice. Analysis of the study suggested that the LncRNA HOTTIP downregulated the miR-214 to increase KPNA3 expression, enhancing CRC resistance to mitomycin [50]. The HSP 40 family protein DNAJB8 could upregulate MDR1 to promote CRC resistance to oxaliplatin by inhibiting the ubiquitinated degradation of TP53 [51]. These studies suggested numerous promising molecular targets. Taking EVs or molecular targets as a starting point, it is a very good prospect to overcome drug resistance by interfering with the secretion and uptake of these EVs or blocking their interaction with the target.

### Extracellular vesicles in drug resistance transfer

EVs can enter recipient cells by horizontally transferring the specific biologically active substances, which might alter the gene expression and transcriptional regulatory programs of recipient cells as well as promote the expression of drug-resistant active molecules. A recent study demonstrated that the addition of CEXs isolated from resistant LS174T cells to the medium of sensitive LS174T cells increased the expression levels of nuclear factor erythroid 2-related factor 2 (Nrf2) and P-glycoprotein (P-gp), leading to the development of drug resistance in the sensitive LS174T cells [52]. Similarly, Zhang and his team isolated CEXs from 5-Fluorouracil (5-FU) -resistant and sensitive cell lines (RKO), which were then co-cultured with 5-FU-sensitive cells. These CEXs obtained from resistant CRC cell lines could significantly enhance the survival of CRC cells in a 5-FU-containing medium. Proteomics and Western blot analyses further confirmed that GSTP1 and p-STAT3 (Tyr705) were highly enriched in CEXs [53]. Moreover, the adriamycin-resistant breast cancer cells secreted exosomes, which caused drug resistance by directly transferring the resistance-associated protein P-gp to sensitive cells [54]. However, the studies, reporting the direct transfer of drug-resistant proteins from EVs to CRC cells, are limited.

### Extracellular vesicles and colorectal cancer microenvironment

The development of CRC resistance is correlated with CRC cells and is supported by their surrounding microenvironment. The activation of CAFs is a hallmark in TME, modulates the basic characteristics of CRC cells, and produces stromal structures, which facilitate CRC growth [55]. A study, investigating the methotrexate (MTX) resistance in CRC cells, identified the differential expression of HEPH, caudal‐related homeobox (CDX) 2, and miR-24-3p in CRC using bioinformatics analysis. The miR-24-3p was carried by CAF-derived exosomes and taken up by CRC cells, resulting in the upregulation of HEPH by targeting CDX2 and accelerating the resistance of CRC cells to MTX [56].

The division between aerobic and hypoxic zones is another characteristic of TME. In general, CRC growth is often disproportionate to the rate of angiogenesis, which leaves CRC cells distant from blood vessels in a state of hypoxia. During the doxorubicin (DOX) treatment in the aerobic/hypoxic TME model, CEXs secreted by aerobic C26 cells developed resistance in the naive hypoxic C26 and RAW 264.7 cells to the cytotoxic effects of DOX. These results were related to the high levels of HIF-1α and the B-cell lymphoma-extra-large anti-apoptotic protein (Bcl-xL), mediating the anti-apoptotic response of recipient cells [57]. Furthermore, EVs can modulate the metabolic reprogramming of CRC cells, creating an acidic microenvironment, which reduces the absorption and efficacy of weakly basic chemotherapeutic drugs, such as anthracyclines, anthraquinones, and vinca alkaloids [58].

In conclusion, the mechanisms of CRC chemoresistance are complex, involving multiple dimensions. EVs can transfer a variety of drug resistance-related active substances to regulate chemoresistance in CRC cells through multiple signaling pathways. How will future studies improve drug sensitivity in drug-resistant CRC? EVs, as an entry point, might be a good choice. Further studies should elucidate the biological functions of EVs and mechanisms mediating drug resistance in CRC. This might facilitate the development of novel target drugs and the transformation of combination drugs. Moreover, EVs might also be a potential next-generation drug delivery system.

## Extracellular vesicles: a novel drug delivery platform

Considering the effects of complex TME around CRC, the safe and effective delivery of drugs into CRC cells is one of the biggest challenges at present. In the past few decades, synthetic nanomaterials-based drug delivery systems have made great progress in targeted CRC therapy. However, these nanocarriers have still certain limitations and do not show satisfactory results.

Recently, in terms of drug delivery efficiency and enhanced therapeutic efficacy, natural carriers have shown surprising results. As compared to the traditional nanoparticles (NPs), EVs have great advantages in biocompatibility, evasion of phagocytic clearance, and intrinsic homing ability and might serve as a new generation of drug delivery systems. However, prior to using EVs against CRC, they should be processed first.

The preparation and bioactivity evaluation of EVs currently rely heavily on effective isolation and purification methods. Depending on the size and properties of EVs, various separation methods can be used to separate EVs from biological fluids. The commonly used methods include centrifugation techniques (differential centrifugation, density-gradient centrifugation, sucrose buffered centrifugation), affinity capture, gel-permeation chromatography, membrane filtration, precipitation and microfluidic devices [8]. Centrifugation techniques are commonly used to separate EVs using centrifugal force. They have the advantages of easy operation and high purity of EVs, and are considered the “gold standard” for EVs separation [59]. The affinity capture method uses the immunoaffinity interactions of EV surface proteins to isolate and purify them. This immunoaffinity interaction-based isolation method allows the isolation of different EV subpopulations secreted by the specified cell type [59]. However, centrifugation techniques and affinity capture are unlikely to be used for large-scale applications due to their time-consuming and costly drawbacks [60]. Gel-permeation chromatography and membrane filtration methods are used for the separation and purification of EVs based on differences in their sizes. Membrane filtration is often used as the first step in the separation of EVs and has relatively rapid separation efficiency; however, the filters are prone to clogging. Gel-permeation chromatography can isolate EVs with high purity; moreover, automated collection methods have been developed with potential translational applications. On the other hand, using this method, it is difficult to isolate fractions of sizes similar to EVs, such as lipoproteins [8, 61]. The precipitation technique relies mainly on polymers to precipitate large amounts of EVs; however, the obtained EVs are of low purity and can be used as a means of EV concentration. Microfluidic devices are based on the full integration of EVs size separation, immunoaffinity separation and dynamic separation, which have the advantages of cost-effectiveness, speed and high purity, but they require complex separation devices [59, 60]. Although these separation methods can obtain a certain quality of EVs, unfortunately, there is no method that can completely separate EVs from biofluids. It is only possible to improve the purity of EVs and reduce the proportion of impurities as much as possible. For this reason, based on the heterogeneity of EVs, it is necessary to combine multiple separation methods to isolate the target EVs.

**Fig. 5:**
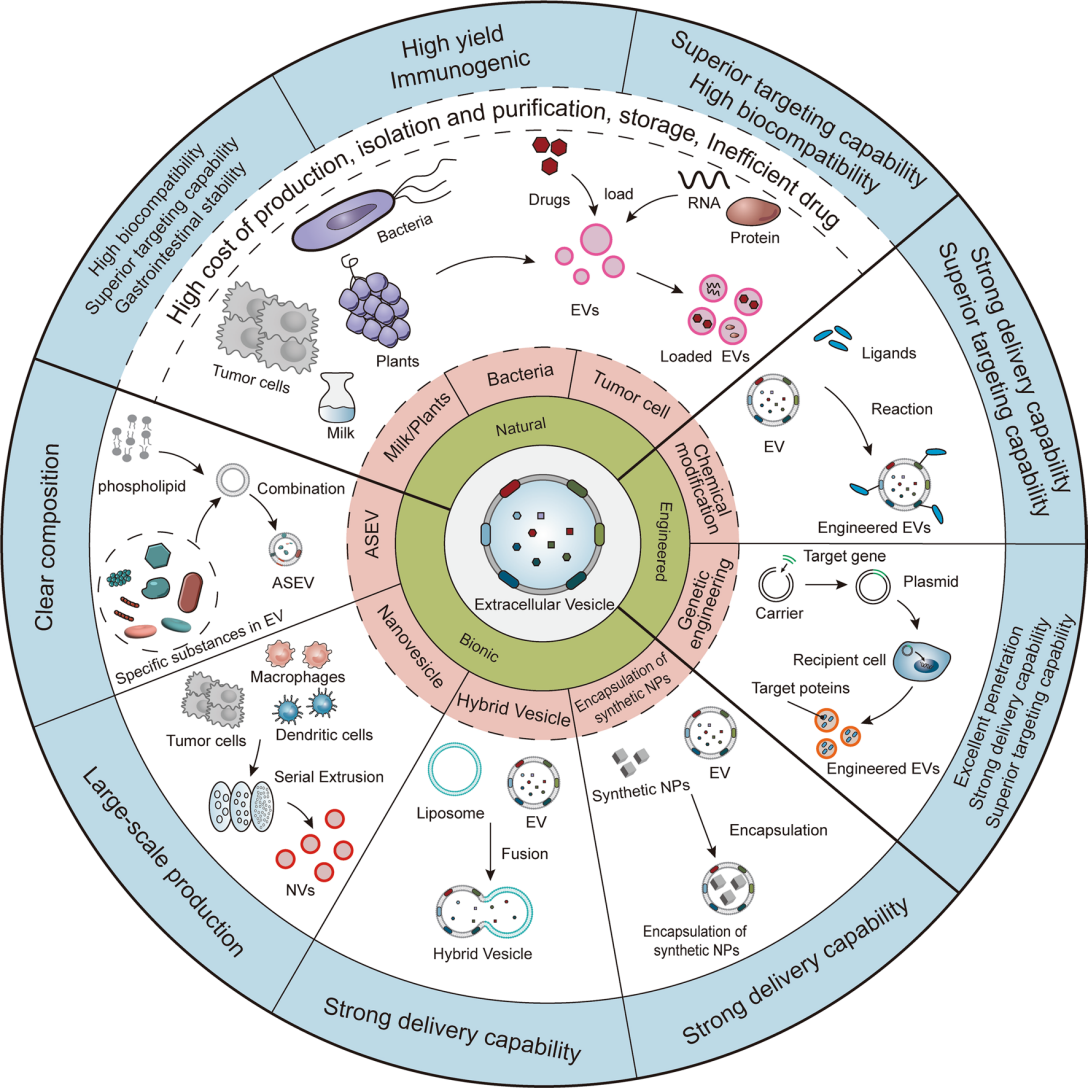
Novel CRC treatment strategy based on EVs

This section summarizes the developments and applications of EVs obtained from different sources as drug delivery systems (Fig. 5).

### Native extracellular vesicles

Currently, the EVs obtained from a variety of cells have been used as drug delivery systems in CRC studies. The lipid and protein composition of EVs depends on the parental cells, and the EVs obtained from different sources might have different activities in terms of homing properties and cancer progression. Therefore, it is necessary to carefully explore the biological properties of each EV and evaluate their pros and cons as drug carriers.

#### Colorectal cancer cell-derived extracellular vesicles

The components of CEXs are similar to those in the CRC cell membranes. Therefore, using CEX as a "porter" for transporting therapeutic drugs might improve their CRC specificity. Recently, Phuong and his team investigated that the exosomes derived from breast and CRC cells enhanced the uptake of nano-amorphous aspirin through clathrin-dependent and independent endocytosis pathways and efficiently delivered aspirin to the tumor sites in vivo [62]. Van et al. isolated EVs from CT26 colon cancer cells and 4T1 murine breast cancer cells and loaded them with DOX by electroporation. The results showed excellent biocompatibility and the ability to target CRC cells [63]. Interestingly, the CT26-derived EVs were preferentially taken up by parental cells and showed robust penetrating properties as compared to 4T1 cell-derived EVs [63]. This just hints at the targeting potential of EVs as drug delivery systems.

Due to the easy RNA degradation, fast clearance, and immune response induction, EVs might also be used in CRC gene therapy [64]. Maryam Hosseini et al. isolated CEXs from CT26 and loaded them with miR-34a mimics. The CEX-miR-34a reduced the invasion, angiogenesis, and immune evasion-related gene expressions in CRC and prolonged the survival of mice with colon cancer. Notably, the mice, receiving CEXs alone, exhibited certain oncogenic gene expressions; however, the IL-17A secretion levels in draining lymph nodes significantly decreased [65]. This suggested that the direct application of CEXs in CRC therapy might face great challenges because the EVs secreted by CRC cells often act as "boosters" and are involved in cell proliferation, induction of angiogenesis, enhancement of invasion and metastasis, immune evasion, and metabolic reprogramming processes [66]. Therefore, ensuring the safety of CEXs for CRC treatment is a current issue, which should be addressed.

#### Bacterial outer membrane vesicles

The bacterial outer membrane vesicles (OMVs) might be a particular choice in immunotherapy. Although the immunogenicity of OMVs might limit their role in cancer therapy as natural drug delivery systems, their small size, immunomodulatory properties, high bacterial production, and enrichment in lymph nodes might lead to their use as vaccine carriers with applications in immunotherapy [67]. Keman and his team used genetic engineering and Plug-and-Display technology to highlight the ClyA fusion protein on the surface of OMVs with different protein capture agent modifications. The results suggested that the OMVs loaded with different tumor antigens inhibited melanoma metastasis and CRC subcutaneous graft growth and might be used as an alternative strategy for cancer immunotherapy [68]. As mentioned earlier, OMVs have the characteristics of parental bacteria and might have a great potential for development. However, further studies on OMVs, obtained from different bacterial sources, are still needed to ensure their safety while being used as vectors.

#### Extracellular vesicles in plants and milk

As compared to synthetic NPs, plant EVs (PEVs) have low toxicity, low immunogenicity, and better cellular uptake and gastrointestinal stability [69]. In a study, a novel oral delivery system based on grapefruit-derived EVs loaded with MTX could target intestinal macrophages, significantly reduce the toxicity of MTX, and improve the therapeutic effects on the mouse colitis models [70].

The milk-derived EVs (MEVs) have similar advantages. The bilayer lipid membrane of MEVs enables them to easily cross the blood–brain barrier and cell membranes, thereby ensuring the target-specific delivery of valuable cargo [71]. A study showed that the orally administered MEVs were stable in the gastrointestinal tract and reduced primary tumor burden in mouse models of CRC and breast cancer. Interestingly, MEVs could aggravate tumor metastasis in the mouse models of breast and pancreatic cancer, while oral administration of MEVs after primary tumor resection could attenuate the metastatic effects of breast cancer cells. Further proteomic and biochemical analyses revealed that MEVs could exert their effects by inducing the cancer cells' senescence and EMT [72].

It can be found that despite the differences in their origins, all the EVs discussed in this study have achieved promising results, highlighting their potential in developing advanced drug delivery systems for CRC treatment. However, the development of native EVs still faces enormous challenges due to the difficulties in large-scale production, isolation and purification, drug-loading efficiency, storage, and uncontrollable heterogeneity among the EVs subpopulations of native EVs [73, 74]. These challenges might greatly hinder the production and clinical applications of EV-related products.

### Artificial extracellular vesicles

Increasing studies have aimed to develop artificial exosomes, such as engineered and biomimetic vesicles through nanobiotechnology. As compared to natural exosomes, these artificial exosomes have higher pharmaceutical acceptability due to solving some of the shortcomings of natural exosomes and easier standardized production rules.

#### Engineered extracellular vesicles

The appropriate surface modification of natural EVs gives them stronger cell-targeting abilities. Genetic engineering and chemical modification are the two commonly used modification strategies. A study investigated the expression levels of miR-1915-3p in a non-tumorigenic intestinal cell line (FHC) by lentiviral transduction. The FHC-derived EVs enhanced the sensitivity of drug-resistant CRC cell lines to oxaliplatin by downregulating the pro-oncogenes *PFKFB3* and *USP2* [75]. Moreover, the combined Her2-mcherry plasmid was transfected into HEK293T cells. The engineered HEK293T cells secreted exosomes, expressing the Her2-LAMP2 fusion protein. The 5-FU and miR-21i were encapsulated by electroporation, forming a co-delivery system. These engineered exosomes effectively reversed drug resistance and significantly enhanced the cytotoxicity of 5-FU-resistant colon cancer cells [76].

In terms of chemical modifications, Elnaz's team covalently attached the carboxylic acid-modified MUC1 aptamer (5TR1) to amine groups on the surface of mesenchymal stem cell-derived exosomes via EDC/NHS chemistry. The modified exosomes showed a higher tumor accumulation and significantly inhibited CRC growth in the CT26 colon adenocarcinoma model [77]. EVs were modified using polyethylene glycols (PEG) to obtain PEGylated EVs, which exhibited a longer life cycle [78]. Alternatively, magnetic NPs (MNPs) and folic acid (FA) have also been prepared into exosome-based hybrid nanostructures (EHNs). EHNs are composed of CEXs, EpCAM-MNPs, and FA and are used to load the anticancer drug DOX. This EHN enhanced the tissue permeability while applying alternating magnetic fields enhanced their CRC cell-killing effects and DOX cytotoxicity [79].

Although the surface modifications of EVs have been successfully performed, the current modification strategies have certain limitations. The prerequisite for genetic engineering techniques requires specific gene fragments for plasmid construction. Due to the huge heterogeneities of EVs, the expression of target protein in all EVs as well as the amount of expression require further verifications. In chemical modifications, ensuring the structural integrity of EVs should be considered. In addition, improving the specificity of site-specific modifications on the surface of EVs without affecting the normal functioning of other EV sites is also a major challenge.

#### Bionic extracellular vesicles

A large proportion of both the natural and engineered EVs are based on natural secretion from donor cells. Large-scale production is difficult to achieve due to their lower yields and higher separation costs. The recently developed artificially synthesized EVs (ASEVs), nanovesicles (NVs), and hybrid vesicles (HVs) can be commercially produced and used for the efficient delivery of drugs to CRC cells.

ASEVs are NPs synthesized based on individual small molecules by linking surface proteins or chemical moieties. The composition of these ASEVs is similar to but clearer than that of natural EVs [80]. Moreover, these ASEVs, along with overcoming the challenges of natural EVs heterogeneity, also overcome the issue of large-scale production. Zhang and his team developed PEGylated liposomes (CTB-sLip) coupled with cholera toxin subunit b protein. These CTB-sLips showed excellent affinity and cellular uptake in both human-derived CRC cell lines, including HCT-116 and HT-29 cells [81]. In another study, immunoliposomes, coated with antibodies to Frizzled 10 protein, were synthesized and loaded with 5-FU. The follow-up results showed that this immunoliposome exhibited good selectivity against CRC as well as improved cytotoxicity of 5-FU [82]. In addition, a bionic magnetic liposome exhibited similar anti-CRC advantages in vitro and in vivo [83]. Despite the great advancements in ASEVs, it is still difficult to fully simulate the natural EVs due to their complex compositions.

NVs are tiny vesicles generated from the donor cells using filters of different pore sizes, microfluidic devices, nitrogen cavitation techniques, and ultrasound or cellular vesicle methods [84]. The components of these NVs are similar to those of the donor cell membrane as well as have similar size, morphology, and protein markers to natural EVs. Notably, the production of NVs increased greatly as compared to natural EVs. Fang et al. prepared “artificially assembled macrophages” by the extrusion of macrophage-derived cell membranes. These NVs showed good CRC targeting with the help of macrophage membranes. They also reversed the immunosuppressive nature of the CRC microenvironment by inhibiting Tregs and promoting the maturation of antigen-presenting cells and achieved a CRC suppression rate of 95.3 ± 2.05% in vivo, due to the combination of photo/chemo/immunotherapy [85]. However, it is noteworthy that the filters used to prepare NVs are prone to clogging.

HVs are usually formed by fusing EVs with other types of membrane structures. A study designed a hybrid therapeutic nanovacuole (hGLV), which was obtained by isolating CD47^+^ gene-engineered exosomes from lentiviral vector-transfected CT26 cells and combining them with drug-loaded thermosensitive liposomes. Follow-up studies showed that CD47^+^ hGLV improved the macrophage-mediated phagocytosis of CRC cells and prolonged circulation time by blocking the CD47 signaling. In addition, hGLV exhibited excellent targeting properties and showed excellent phototherapeutic effects on CRC after laser irradiation. It could also promote the maturation of immature DCs with the help of a co-encapsulated immune adjuvant, thereby triggering a stronger immune response [86].

In addition to the above-mentioned applications, EVs have also been used as "invisible coats" to wrap the existing synthetic NPs. A study was conducted to synthesize a photoactivatable polymer TKPEI-Ce6 using the cross-linked branched polyethyleneimine, reactive oxygen species-sensitive thiacetal, and photosensitizer chlorin e6. Further, a nanomimetic photoactivated silencing EVs (PASEVs) were successfully developed using M1 macrophage-derived EVs wrapped with TKPEI-Ce6 and a small interfering RNA targeting PAK4. PASEVs achieved effective accumulation at CRC sites, and after using laser irradiation, PASEVs promoted immune activation in the CRC microenvironment and exerted a powerful role in killing CRC [87].

In conclusion, biomimetic NPs are highly biocompatible, have limited immunogenicity, and are capable of being produced on a large scale, thereby making them a promising drug delivery vehicle in the future. However, the molecular mechanisms of EVs biogenesis have not been fully understood yet, and there is a lack of unified technical standards. Therefore, further studies are still needed to elucidate the mechanisms of EVs biogenesis, cargo packaging, cell penetration, and regulatory evasion to further amplify the potential of EV-related drug carriers.

## Biomarkers

### Extracellular vesicles and biomarkers

Recently, the use of EVs as biomarkers for the prediction, diagnosis, and presumed prognosis of CRC has shown promising outcomes. EVs can be used as biomarkers for the development of CRC and are summarized in Table 3. A study using real-time quantitative polymerase chain reaction (RT-qPCR) showed a significant decrease in the plasma EV-miR-193a-5p levels in CRC patients. The EV-miR-193a-5p showed an area under the receiver operating characteristic (ROC) curve (AUC) of 0.740 in differentiating CRC from precancerous colorectal adenomas and 0.759 in differentiating CRC from non-cancerous controls. Further analysis showed that miR-193a-5p could target the tumor-associated genes, including CUT-like homeobox 1 and intersectin 1, and inhibit CRC progression. These findings suggested that miR-193a-5p might help in predicting the occurrence and progression of CRC [88].

**Table 3:** EVs as biomarkers in CRC

CRC biomarker	Source	Function	References
miR-193a-5p	Plasma	Targeting tumor-associated genes CUX1 and ITSN1 to inhibit CRC migration and invasion	[88]
miR-320c	Plasma	Involving MET and forming PMNs	[124]
miR-21, miR-92a, miR-222	Serum	Associated with lower overall survival	[90]
miR-181b, miR-193b, miR-195, miR-411	Serum	Effective identification of patients with invasive submucosal CRC at risk of lymph node metastasis in a preoperative setting	[125]
miR-361-3p	Tumor	Targeting TRAF3 activates non-classical NF-κB pathway.Associated with worsening CRC prognosis	[126]
CircLPAR1	Plasma	Reducing the translation of the oncogene BRD4 by binding with eIF3h and inhibiting the METTL3-eIF3h interaction	[89]
SPARC, LRG1	Serum	Associated with CRC sidedness and predicted CRC recurrence	[127]
QSOX1	Plasma	For early diagnosis and non-invasive risk stratification	[128]
CD59, TSPAN9	Plasma	Differentiating between CRC and stage I/II CRC patients	[129]
FGB, β2-GP1	CRC tissue	Diagnosing early CRC	[130]

*CUX1* CUT-like homeobox 1, *ITSN1* Intersectin 1, *MET* Mesenchymal-epithelial transition, *TRAF3* TNF receptor-associated factor 3, *eIF3h* Eukaryotic translation initiation factor 3 subunit h, *METTL3* Methyltransferase-like 3, *SPARC* Secreted protein acidic and cysteine rich, *LRG1* Leucine rich alpha-2-glycoprotein 1, *QSOX1* Quiescin sulfhydryl oxidase 1, *TSPAN59* Tetraspanin 9, *FGB* Fibrinogen beta chain, *β2-GP1* Beta-2-glycoprotein 1

EVs might also provide novel perspectives for the early clinical diagnosis and disease pathogenesis of CRC. The diagnostic performance of exosomal CircLPAR1 in plasma was analyzed by combining the common clinical biomarkers, including CEA and CA19-9. The results demonstrated that the exosomal CircLPAR1 showed improved diagnostic performance (AUC of 0.875) while diagnosing CRC and was correlated with the overall survival of patients [89]. Moreover, CircLPAR1 encapsulated in exosomes exhibited high stability and detectability.

In addition, EVs might also predict the prognosis of CRC patients. A study suggested the superior performance of the serum EVs-associated miRNA-21 and miRNA-92a in diagnosing patients with metastatic CRC as compared to that of carcinoembryonic antigen. Further multivariate Cox analysis confirmed that the higher levels of miRNA-222 were correlated with lower overall survival of patients [90]. These data all implied that EVs might have a strong potential as a novel non-invasive biomarker for the prediction, diagnosis, and presumed prognosis of CRC.

### Detection of biomarkers

Despite the great potential of EVs for liquid biopsy-based prediction, diagnosis, and presumed prognosis of CRC, the detection and characterization of such small molecules with high sensitivity are the major challenges since they are highly heterogeneous and microscopic in nature. Several techniques have been developed to determine the physical properties and biological characteristics of EVs, including transmission electron microscopy, nanoparticle-tracking analysis, Western blotting, ELISA, and high-sensitivity flow cytometry. Transmission electron microscopy is typically used to observe the size and surface characteristics of EVs. Nanoparticle-tracking analysis can be used to determine the size distribution and concentration of EVs; however, this method cannot detect specific subpopulations of EVs [60]. Western blotting is a quantitative or semi-quantitative method for monitoring the levels of characteristic proteins targeted by EVs with small sample sizes [91]. ELISA is used to quantify specific EV-binding proteins based on colorimetric or fluorescent methods, but it requires the separation of EVs from soluble fractions [61]. High-sensitivity flow cytometry classifies the subpopulations of EVs and determines the origin of EVs by simultaneously detecting their light scattering and fluorescence signals. However, efforts are still needed to improve its sensitivity [92]. Among these, ELISA and high-sensitivity flow cytometry are more likely to be used for the detection of EVs as CRC biomarkers in the routine clinical setting due to their reproducibility, low cost, and the advantage of distinguishing EV subgroups.

In addition to the above commonly used assays, studies are continuously being conducted on the development of more accurate, faster, and cost-effective technologies for the detection of biomarkers associated with EVs in CRC. For example, Wei and his team performed immunoassays on circulating EVs (CD9-CD63 and EpCAM-CD63) by developing a single-molecule array technique. The results demonstrated that the performances of the single-molecule array technique gave and Western blotting for EVs expressing EpCAM were similar, and the single-molecule array technique could distinguish between cancerous and non-cancerous plasma samples [93]. Francisco and his team presented a microfluidic amperometric immunosensor assay for detecting the biomarker claudin7 (CLD7). The results showed that the detection range of the detector was 2–1000 pg mL^−1^, and the detection limit of CLD7 could reach 0.1 pg mL^−1^ under optimal conditions [94].

However, it is worth noting that all these methods require the separation and purification of EVs. Moreover, the possibility of multiplex analysis of EVs is also not known yet. This undoubtedly increases the difficulty and complexity of sample processing. To address this issue, Zhang et al. used surface-enhanced Raman spectroscopy nanotags to simultaneously analyze multiple protein biomarkers expressed on the CEXs. As compared to most of the other reported EV detection techniques, such as Western blotting, ELISA, and flow cytometry, the surface-enhanced Raman spectroscopy nanotag analysis eliminated the need for complex separation steps and enabled the possibility of higher multiplex analysis with higher sensitivity. This might greatly simplify the sample preparation process and increase the efficiency of the assay, allowing for instant determination [95].

## Conclusion and prospects

The current studies have demonstrated that EVs are involved in bidirectional crosstalk between CRC cells and recipient cells as well as in compiling the dynamic network of TME, conferring the CRC cells to proliferate, metastasize, and develop drug resistance. Based on abundant evidence, this review described the biological functions of EVs in promoting CRC cell dissemination and developing resistance to therapy by regulating the CRC microenvironment. This intrinsic property might provide a new starting point for the prediction, diagnosis, and treatment of CRC. EVs have been proposed as predictive and diagnostic biomarkers due to their easy isolation from the patient’s body fluids and their potential to highlight CRC. For therapy, investigating the pathways of EVs might potentially lead to identifying novel therapeutic targets. Considering their natural origin, EVs can improve biocompatibility and target penetration. This review also further discussed the therapeutic potential of EVs as delivery systems in CRC.

However, in practical work, there are certain limitations in the separation and purification of EVs, making their large-scale production difficult. EVs have huge heterogeneity, which might result in the uncontrollability of their clinical applications. The application of bionic technology might have good prospects for the development of EVs; however, a complete set of standardized rules to guide the production and evaluation of bionic EVs should be formed. Additionally, the available technologies for EVs detection are not yet mature, which might not be conducive to their clinical implementation. Therefore, the biogenesis process of EVs and mechanisms of their participation in cargo sorting to regulate CRC cells should be further explored. This might help in identifying a specific way to solve these defects. In conclusion, EVs are highly potential delivery media in TME; however, further research is needed to further explore them.

## Data Availability

Not applicable.

## Abbreviations

CRC: Colorectal cancer
EVs: Extracellular vesicles
HSP: Heat shock proteins
ICAM: Intercellular cell adhesion molecule
MVBs: Multivesicular bodies
ESCRT: Endosomal sorting complex required for transport
MHC: Major histocompatibility complex
ILVs: Intraluminal vesicles
TME: Tumor microenvironment
CEXs: Colorectal cancer cell-derived exosomes
CAFs: Cancer-associated fibroblasts
PTEN: Phosphatase and tensin homolog
SOCS3: Suppressor of cytokine signaling 3
EMT: Epithelial-to-mesenchymal transition
TGF-β: Transforming growth factor-β
hTERT: Human telomerase reverse transcriptase
PDCD4: Programmed cell death 4
SOCS1: Suppressor of cytokine signaling 1
ZBTB2: Zinc finger and BTB domain-containing 2
ITGBL1: Integrin β-like 1
HAPLN1: Hyaluronan and proteoglycan link protein -1
ECM: Extracellular matrix
MAGP: Microfibril-associated glycoprotein
PMNs: Pre-metastatic niches
VE-Cad: Vascular endothelial adhesion protein
HSCs: Hepatic stellate cells
KCs: Kupffer cells
MMP: Matrix metalloproteinase
DCs: Dendritic cells
NK cells: Natural killer cells
TAMs: Tumor-associated macrophages
MDSCs: Myeloid-derived suppressor cells
ICIs: Immune checkpoint inhibitors
HuR: Human antigen R
Nrf2: Nuclear factor erythroid 2-related factor 2
P-gp: P-glycoprotein
5-FU: 5-Fluorouracil
MTX: Methotrexate
CDX2: Caudal‐related homeobox 2
DOX: Doxorubicin
Bcl-xL: B-cell lymphoma-extra-large anti-apoptotic protein
NPs: Nanoparticles
OMVs: Outer membrane vesicles
PEVs: Plant EVs
MEVs: Milk-derived EVs
PEG: Polyethylene glycols
MNPs: Magnetic NPs
FA: Folic acid
EHNs: Exosome-based hybrid nanostructures
ASEVs: Artificially synthesized EVs
NVs: Nanovesicles
HVs: Hybrid vesicles
PASEVs: Photoactivated silencing EVs
RT-qPCR: Real-time quantitative polymerase chain reaction
ROC: Receiver operating characteristic curve
CLD7: Claudin7
